# Complete Genome Sequence of “*Candidatus* Liberibacter africanus,” a Bacterium Associated with Citrus Huanglongbing

**DOI:** 10.1128/genomeA.00733-15

**Published:** 2015-07-16

**Authors:** Hong Lin, Gerhard Pietersen, Cliff Han, David Alan Read, Binghai Lou, Goutam Gupta, Edwin L. Civerolo

**Affiliations:** a USDA-ARS San Joaquin Valley Agricultural Sciences Center, Parlier, California, USA; b University of Pretoria, Pretoria, South Africa; c Los Alamos National Laboratory, Los Alamos, New Mexico, USA; d Guangxi Citrus Research Institute, Guilin, China

## Abstract

We report here the complete genome sequence of “*Candidatus* Liberibacter africanus” strain PTSAPSY. The 1,192,232-bp genome with 34.5% G+C content comprises 1,017 open reading frames, 44 tRNAs, and three complete rRNAs in a circular chromosome.

## GENOME ANNOUNCEMENT

Huanglongbing (HLB), also known as greening disease, is one of the most devastating diseases of citrus in the world (1). The disease is associated with three phloem-restricted insect-transmitted, Gram-negative, and fastidious species of alphaproteobacteria in the genus “*Candidatus* Liberibacter.” The name of each species was based on its presumptive origin; “*Candidatus* Liberibacter asiaticus” is believed to have originated in Asia, “*Ca.* Liberibacter americanus” in the Americas, and “*Ca.* Liberibacter africanus” in Africa (1–4). Among these three species, “*Ca.* Liberibacter asiaticus” is the most widely distributed and transmitted by the Asian citrus psyllid *Diaphorina citris* worldwide, while “*Ca.* Liberibacter africanus” is found only in Africa and the Mascarene Islands and is naturally transmitted by the psyllid *Trioza erytreae*. In contrast to “*Ca.* Liberibacter asiaticus” and “*Ca.* Liberibacter americanus,” “*Ca.* Liberibacter africanus” is heat sensitive and often suppressed at elevated temperatures of >30°C (1). In South Africa, a disease was observed as a yellow shoot or greening resembling mineral toxicity in 1928 and was confirmed to be a biotic disease in 1965 (1). While still a serious disease prevalent in many parts of South Africa (5), citriculture has flourished in spite of greening due to efficient vector control, inoculum removal strategies, and the milder nature of the disease caused by “*Ca.* Liberibacter africanus.”

We completed the genome sequence of a “*Ca.* Liberibacter africanus” strain, PTSAPSY. Genomic DNA was isolated from individual psyllids (*T. erytreae*) collected from Pretoria, South Africa. DNA samples containing higher titers of “*Ca.* Liberibacter africanus,” estimated by real-time PCR, were selected for whole-genome amplification. Amplified “*Ca.* Liberibacter africanus” genomic DNA was used to construct a sequencing library. The complete genome sequence of the strain was obtained by an Illumina HiSeq 2000 sequencing run with a 300-bp paired-end library, which achieved an average coverage of 60 to 80×. *De novo* assembly was conducted using the Velvet assembler version 1.1.04. “*Ca.* Liberibacter africanus” contigs were identified via BLASTn and BLASTx analyses against genomes of “*Ca.* Liberibacter asiaticus” (6), “*Ca.* Liberibacter americanus” (7, 8), “*Ca.* Liberibacter solanacearum” (9), and the phylogenetically related bacteria *Agrobacterium tumefaciens* and *Sinorhizobium meliloti*. All identified contigs were reconfirmed by PCR. The contig gaps were then closed by PCR-based primer walking (9), and gap-closing PCR products were resequenced using the Sanger sequencing method. Final annotation was performed by using the NCBI Prokaryotic Genomes Annotation Pipeline (PGAP) (http://www.ncbi.nlm.nih.gov/genomes/static/Pipeline.html).

The genome of strain PTSAPSY comprises 1,192,232 nucleotides, 34.5% G+C content, 1,017 predicted coding sequences, 44 tRNAs, three complete copies of rRNA genes (16S, 23S, and 5S), and 279 hypothetical genes. Like “*Ca.* Liberibacter americanus” and “*Ca.* Liberibacter solanacearum,” two tandemly aligned prophage segments were identified in the “*Ca.* Liberibacter africanus” genome. The average G+C content in this prophage region is 42.1%, deviating significantly from that of the core genome. Genome information will contribute to a deeper understanding of the genomic evolution of liberibacters, permit comparative genomic analysis with other “*Ca.* Liberibacter” species, and may shed light on the molecular mechanisms associated with pathogenicity.

### Nucleotide sequence accession number.

The completed genome sequence of “*Ca.* Liberibacter africanus” strain PTSAPSY has been deposited in the GenBank database under the assigned accession no. CP004021. The version described in this paper is the first version.
